# Measuring impostor phenomenon in healthcare simulation educators: a validation of the clance impostor phenomenon scale and leary impostorism scale

**DOI:** 10.1186/s12909-022-03190-4

**Published:** 2022-03-03

**Authors:** Kirsty J. Freeman, Stephen Houghton, Sandra E. Carr, Debra Nestel

**Affiliations:** 1 Duke NUS Medical School, Singapore, Singapore; 2 Graduate School of Education, University of Western Australia, Perth, Australia; 3 Health Professions Education, School of Allied Health, The University of Western Australia, Perth, Australia; 4 School of Clinical Sciences, Monash University, Melbourne, Australia; 5 Department of Surgery, University of Melbourne, Melbourne, Australia

**Keywords:** Impostor Phenomenon, Healthcare Simulation Educators, Clance Impostor Phenomenon Scale, Leary Impostor Scale, Exploratory Factors Analysis

## Abstract

**Background:**

Impostor phenomenon is a term used to describe feelings of intellectual and professional fraudulence. The Clance Impostor Phenomenon Scale and the Leary Impostorism Scale are two self-report measures used to determine whether an individual experiences impostor phenomenon. This study examined the psychometric properties of both measures in healthcare simulation educators.

**Methods:**

The study sample comprised 148 educators, 114 (77%) females, 34 (23%) males, who completed an online version of each instrument. Exploratory factor analysis was used to examine the factor structure of the Clance Impostor Phenomenon Scale and the Leary Impostorism Scale.

**Results:**

Exploratory factor analysis revealed that for both instruments a one-factor solution best fit the data, suggesting all items in both measures fit onto a single theoretical construct.

Both instruments demonstrated high internal reliability, with the Cronbach’s alpha for the Clance Impostor Phenomenon Scale being α = .96 and the Leary Impostorism Scale α = .95.

**Conclusions:**

This study suggests that impostor phenomenon as measured by the Clance Impostor Phenomenon Scale and the Leary Impostorism Scale is a unidimensional construct among healthcare simulation educators. With a growing interest in impostor phenomenon, the present findings will assist researchers to evaluate the phenomenon in healthcare settings.

## Background

Described as an individual’s persistent belief that they lack intelligence, skills or competence, and are not worthy of success, [1–3] the term impostor phenomenon (IP) was first introduced by Clance and Imes [2]. Their study of 150 highly successful women found that “despite outstanding academic and professional accomplishments, women who experience the impostor phenomenon persist in believing that they are really not bright and have fooled anyone who thinks otherwise” [2]. Over the last 40 years research has shown that IP is experienced by individuals irrespective of gender, profession or cultural background [4–8].

Harvey and Katz suggested that nearly 70% of successful people have experienced IP in their working life [3]. Studies of IP in healthcare professionals has been focused on those entering the workforce for the first time, [9, 10] with only a few studies investigating the phenomenon in the current workforce [11–13]. The existence and impact of IP in the healthcare simulation educators is unknown. IP exists on a continuum from occasional feelings of inadequacy to constant feelings of being exposed as a fraud [1]. The more frequently IP is experienced the greater the likelihood of negative effects. IP has been reported to negatively impact job satisfaction, [14] increase emotional exhaustion, [15] and lead to burnout [16]. To enable faculty developers to mitigate the negative impact of IP in the healthcare simulation educator community we must first investigate the prevalence.

The increasing level of interest in IP has seen the development of several self-report instruments to measure IP, including the Clance Impostor Phenomenon Scale (CIPS), [17] Harvey Impostor Scale, [18] Perceived Fraudulence Scale, [19] and Leary Impostorism Scale (LIS) [20]. The LIS is a 7 item unidimensional instrument measuring a person’s sense of being an impostor or fraud [20]. The authors have reported high inter-item reliability (α = 0.87). The 20 item CIPS is reported to be the most commonly used scale by those researching IP, with Cronbach alphas ranging from 0.85 to 0.96 [21]. Chrisman et al. reported that the CIPS comprises three factors – luck, fake and discount [22]. Brauer and Wolf also reported three factors in their EFA of the German version of the CIPS [23]. Subsequent studies suggest one, two and three-factor models best explain the factor structure of the instrument [6, 7, 24, 25]. For researchers investigating IP in healthcare simulation educators the use of an instrument that has been validated within a related context is important. This together with the limited number of studies reporting the use of the 7-item LIS offers a clear rationale for further examination and validation of the instrument. The aim of this study is to examine the psychometric properties of the CIPS and LIS, and provide evidence for their validity within the healthcare simulation population.

## Method

### Participants and procedure

The sample comprised 148 healthcare simulation educators, 114 (77%) females and 34 (23%) males. Of the respondents, 86 (58%) were aged 40 to 55 years, 37 (25%) 56 to 74 years and 25 (17%) 24 to 39 years. Responses were received from respondents in nine countries, with the majority currently working in the United States of America (61%), followed by Australia (22%), and United Kingdom (7%), with Canada, Denmark, Portugal, Singapore, Thailand and Turkey comprising the remaining 10%.

The study was approved by the Human Ethics Research Office of The University of Western Australia (RA/4/20/5061). An invitation to participate in the study was distributed via the international simulation community through SimConnect, the online community platform of the Society for Simulation in Healthcare, based in the USA (*n* = 4000); the Australian based National Health Education and Training in Simulation (NHET-Sim) community (*n* = 5000); and the WA Simulation in Healthcare Alliance (*n* = 20), based in Western Australia. A cover letter informed all potential respondents their participation was voluntary and that responses were anonymous. Those who agreed to take part were given a link to an online form, where they were introduced to the research objectives. They were then provided with instructions on completing the anonymous questionnaires, subject to confirming their informed consent. All respondents provided demographic information, such as age, gender, and geographical location, along with other information. They then completed the CIPS and the LIS. No incentives were provided.

### Measurement of impostor phenomenon

The psychometric properties of the CIPS and LIS were examined by establishing the respective factor structures from a sample of 148 healthcare simulation educators. An Exploratory Factor Analysis (EFA) was employed to establish which latent variables optimally summarised the observed variables on each of the measures.

### Analyses

Prior to performing the EFA, the suitability of the data for factor analysis was assessed on the basis on three criteria: (a) a visual inspection of the data matrix for correlations in excess of 0.30, [26] (b) Bartlett’s test of sphericity [27] and (c) Kaiser–Meyer–Olkin’s measure of sampling adequacy [28]. After assessing the suitability, the 20 items of the CIPS, and the 7 items of the LIS were subjected to maximum likelihood (ML) factor analysis with (orthogonal) varimax rotation. According to Kline, loadings of 0.3 and above are regarded as significant with a minimum sample of 100 participants [29]. The number of factors to be retained for interpretation were then determined by six criteria: 1) Kaiser-Guttman’s eigenvalue of greater than 1.0 rule; 2) Cattell’s scree test; 3) Horn’s parallel analysis; 4) the cumulative percentage of variance criterion; 5) the evaluation of factor loading patterns; and 6) the interpretability criterion of factor loading [30–34]. Analyses were conducted using IBM SPSS version 27.

## Results

### CIPS

The correlation matrix revealed the presence of a substantial number of correlation coefficients above 0.3, indicating some underlying relationship among the variables and therefore the suitability of the correlation matrix for factor analysis. Bartlett’s test of sphericity [22] (χ^2^ = 2232, *df* = 190, *p* < 0.001), supported the factorability of the matrix, while Kaiser–Meyer–Olkin’s measure of sampling adequacy [28] (0.95), suggested sufficient common variance among the observed variables for factor analysis. Descriptive statistics for the CIPS are shown in Table 1.

**Table 1:** Descriptive statistics and CITC of the CIPS 20 items (*N* = 148)

CIPS Items	Mean	SD	CITC
1—I have often succeeded on a test or task even though I was afraid that I would not do well before I undertook the task	3.52	1.13	.58
2—I can give the impression that I’m more competent than I really am	2.98	1.17	.61
3—I avoid evaluations if possible and have a dread of others evaluating me	2.26	1.05	.49
4—When people praise me for something I’ve accomplished, I’m afraid I won’t be able to live up to their expectations of me in the future	2.70	1.21	.82
5—I sometimes think I obtained my present position or gained my present success because I happened to be in the right place at the right time or knew the right people	2.97	1.45	.73
6—I’m afraid people important to me may find out that I’m not as capable as they think I am	2.53	1.27	.81
7—I tend to remember the incidents in which I have not done my best more than those times I have done my best	3.24	1.26	.73
8—I rarely do a project or task as well as I’d like to do it	2.71	1.21	.66
9—Sometimes I feel or believe that my success in my life or in my job has been the result of some kind of error	2.00	1.19	.75
10—It’s hard for me to accept compliments or praise about my intelligence or accomplishments	3.26	1.10	.68
11—At times, I feel my success has been due to some kind of luck	2.43	1.19	.77
12—I’m disappointed at times in my present accomplishments and think I should have accomplished much more	2.98	1.18	.59
13—Sometimes I’m afraid others will discover how much knowledge or ability I really lack	2.43	1.15	.82
14—I’m often afraid that I may fail at a new assignment or undertaking even though I generally do well at what I attempt	2.79	1.14	.81
15—When I’ve succeeded at something and received recognition for my accomplishments, I have doubts that I can keep repeating that success	2.55	1.18	.84
16—If I receive a great deal of praise and recognition for something I’ve accomplished, I tend to discount the importance of what I’ve done	3.20	1.17	.71
17—I often compare my ability to those around me and think they may be more intelligent than I am	3.06	1.32	.75
18—I often worry about not succeeding with a project or examination, even though others around me have considerable confidence that I will do well	2.94	1.22	.79
19—If I’m going to receive a promotion or gain recognition of some kind, I hesitate to tell others until it is an accomplished fact	3.60	1.20	.63
20—I feel bad and discouraged if I’m not “the best” or at least “very special” in situations that involve achievement	2.91	1.23	.59

Response options ranged from 1 = *not at all true* to 5 = *very true*. *CITC* – corrected item-total correlations; *CIPS* = Clance Impostor Phenomenon Scale

A maximum likelihood (ML) factor analysis with (orthogonal) varimax was conducted. ML factor analysis extracts a set of factors by successive factoring, each of which in turn explains as much variance as possible in the population correlation matrix, as estimated from the sample correlation matrix [29]. ML provides statistical significance testing of each factor as it is extracted, [34] and varimax is the preferred method of rotation when the research goal is item reduction and where data will be subsequently used in multivariate analysis [33].

An unrestricted ML factor analysis with varimax rotation revealed the presence of two possible factors with eigenvalues exceeding 1.0, explaining 55.69% and 5.76%, of the variance, respectively. The screeplot suggested a probable one-factor solution in comparison with the two-factor solution derived with the eigenvalue rule. The first factor comprised of 12 items and the second 8 items. As shown in Table 2, of the 20 items, 11 cross loaded with values on both factors greater than 0.39, with some having cross loadings on both factors exceeding this.

**Table 2:** Unrestricted factor-loading matrix

	Factor	
	1	2
CIPS Items		
Q18	**.856**	.301
Q14	**.783**	.389
Q17	**.731**	.361
Q15	**.681**	.539
Q19	**.585**	.293
Q12	**.551**	.306
Q1	**.533**	.293
Q7	**.529**	.511
Q16	**.527**	.475
Q10	**.521**	.450
Q8	**.510**	.439
Q20	**.501**	.332
Q11	.348	**.793**
Q9	.321	**.784**
Q5	.317	**.755**
Q6	.456	**.744**
Q13	.519	**.695**
Q4	.571	**.606**
Q2	.336	**.550**
Q3	.334	**.371**

Loadings highlighted in bold indicate the factor on which the item was placed

Given the cross-loadings the number of factors to extract was then manually specified at three and four, but in each case the factor solution consisted of multiple cross loadings, items not loading on any factor, and a single item factor being derived. In each of these analyses the iterative removal of items did not resolve the problems.

The factor analysis was re-run with one factor specified and this revealed a one factor solution, explaining 55.69%, of the variance. As can be seen in Table 3 all variables had loadings above 0.30 and all loaded on the single factor.

**Table 3:** One factor specified factor-loading matrix

CIPS ITEMS	
Q15	.867
Q13	.856
Q6	.844
Q4	.836
Q14	.823
Q18	.808
Q11	.795
Q9	.772
Q17	.770
Q5	.749
Q7	.735
Q16	.715
Q10	.693
Q8	.673
Q2	.627
Q19	.625
Q12	.607
Q20	.592
Q1	.588
Q3	.502

Extraction Method: Maximum Likelihood

A parallel analysis [32] was conducted with a randomly generated data set (20 variables, *N* = 148, 100 to 1000 replications was run) using a Monte Carlo PCA for parallel analysis [34]. The results indicated that for one factor, the eigenvalues from the sample in this study (11.138) exceeded the corresponding eigenvalues (PCA max = 1.73; 100 to 1,000 replications). This further suggests that all items on the CIPS were assessing one underlying dimension.

The cumulative percent of the one-factor solution of the 20 CIPS items accounted for 55.69%, which is above the minimum level of 30%. The interpretability of the items and factor loadings was impostor phenomenon. Cronbach’s alpha for the CIPS was α = 0.96, indicating a high degree of internal consistency.

### The LIS

The same EFA procedure used with the CIPS was applied to the seven items of the LIS. The correlation matrix revealed the presence of a substantial number of correlation coefficients above 0.3. Bartlett’s test (χ2 = 885, *df* = 21, *p* < 0.001) supported the factorability of the correlation matrix, while the MSA (0.91) suggested sufficient common variance among the observed variables for factor analysis.

An unrestricted maximum likelihood (ML) factor analysis with (orthogonal) varimax rotation revealed the presence of one possible factor with an eigenvalue exceeding 1.0, and explaining 73.4% of the variance. Table 4 shows the mean and standard deviation for each of the items.

**Table 4:** Descriptive statistics for the 7 item LIS and CITC (*N* = 148)

Item	Mean	SD	Corrected Item-Total Correlation
1—Sometimes I am afraid I will be discovered for who I really am	2.17	1.097	.826
2—I tend to feel like a phony	1.99	1.085	.837
3—I'm afraid people important to me may find out that I'm not as capable as they think I am	2.39	1.248	.845
4—In some situations I feel like an imposter	2.47	1.209	.817
5—Sometimes I’m afraid others will discover how much knowledge or ability I really lack	2.39	1.187	.864
6—In some situations I feel like a "great pretender"; that is, I'm not as genuine as others think I am	2.03	1.112	.819
7—In some situations I act like an imposter	1.74	.971	.591

Response options ranged from 1 = *is not characteristic at all of me* to 5 = *extremely of me*. *CITC* corrected item-total correlations

An inspection of the screeplot for the LIS revealed a probable one-factor solution and the cumulative percent of the one-factor solution of the seven LIS items accounted for 73.4% of the variance. All items had loadings above 0.30 and all loaded on the single factor (see Table 5). Item 7 cross-loaded but removal of this item did not improve the factor solution or the reliability of the scale that comprised distinctively of items measuring impostor phenomenon.

**Table 5:** Unrestricted factor-loading matrix

	Factor	
	1	2
Q1	**.911**	-.122
Q2	**.898**	-.195
Q3	**.854**	.023
Q4	**.851**	-.038
Q5	**.846**	.304
Q6	**.839**	.030
Q7	**.610**	.370

Extraction Method: Maximum Likelihood

A parallel analysis (7 variables, *N* = 148, 50 to 1000 replications conducted) indicated that for one factor, the eigenvalue (5.138) from the sample in this study exceeded the corresponding eigenvalues (PCA max = 1.323; 50 to 1,000 replications) obtained from the randomly generated data set of the same size. Cronbach’s alpha for the LIS was α = 0.94, suggesting that it has a high level of internal consistency. This indicates that the LIS functioned as a relatively homogeneous scale in this sample.

With the EFA supporting a one-factor solution for both instruments, a Pearson correlation coefficient was conducted to assess the relationship between the total scores on the LIS and the CIPS. There was a significant positive correlation between the total scores of the measures, *r* = 0.828, *n* = 148, *p* =  < 0.001.

## Discussion

The CIPS and LIS are two instruments frequently used to measure the construct of impostor phenomenon, the former being the most popular. Previous studies have reported inconsistent findings, however, with regard to their psychometric properties. Therefore, this present study sought to establish their factorial structure and internal reliability.

The EFAs conducted supported a one factor model for both the 20-items CIPS and the 7-items LIS. Both instruments also produced high internal reliabilities. Previous studies have reported one, two, three and four factor solutions identifying subscales such as luck, fear, fake, and discount. When confirmatory factor analysis has been used for the CIPS, only 16 items have been included and it has been unclear which items load onto the factors. Mak et al. recently noted that the CIPS does not measure subscale characteristics [21] Therefore, optimum factor structures have not been clear. By conducting a rigorous EFA the present findings indicate both measures are unidimensional, which is consistent with Jöstl et al., [7] and Simon and Choi, [24] who report a single factor best explains the structure of the CIPS. Similarly, previous research shows the 7 item LIS is also a unidimensional measure of impostorism [35]. A significant positive correlation was established between the total scores of the CIPS and LIS, suggesting that researchers investigating IP in healthcare simulation educators could utilise the shorter 7-item LIS.

## Conclusion

This study suggests that impostor phenomenon, as measured by the CIPS and LIS, is a unidimensional construct, and that both the CIPS and LIS have sound psychometric properties for measuring IP among healthcare simulation educators. With the increasing interest in IP globally, these findings will increase the scientific community’s confidence in measuring the construct using CIPS and LIS.

## Data Availability

The datasets used and/or analysed during the current study are available from the corresponding author on reasonable request.

## Abbreviations

IP: Impostor phenomenon
CIPS: Clance Impostor Phenomenon Scale
LIS: Leary Impostorism Scale
EFA: Exploratory Factor Analysis
ML: Maximum likelihood
MSA: Measurement statistical analysis
